# Global Ubiquitome Profiling Revealed the Roles of Ubiquitinated Proteins in Metabolic Pathways of Tea Leaves in Responding to Drought Stress

**DOI:** 10.1038/s41598-019-41041-3

**Published:** 2019-03-12

**Authors:** Hui Xie, Yu Wang, Yiqian Ding, Chen Qiu, Litao Sun, Zhongshuai Gai, Honglian Gu, Zhaotang Ding

**Affiliations:** 1 Tea Research Institute, Qingdao Agricultural University, Qingdao, 266109 China; 2 College of Life Science, Yantai University, Yantai, Shandong, 264005 China

## Abstract

Drought stress often affects the expression of genes and proteins in tea plants. However, the global profiling of ubiquitinated (Kub) proteins in tea plants remains unearthed. Here, we performed the ubiquitome in tea leaves under drought stress using antibody-based affinity enrichment coupled with LC-MS/MS analysis. In total, 1,409 lysine Kub sites in 781 proteins were identified, of which 14 sites in 12 proteins were up-regulated and 123 sites in 91 proteins down-regulated under drought stress. The identified Kub proteins were mainly located in the cytosol (31%), chloroplast (27%) and nuclear (19%). Moreover, 5 conserved motifs in EK^ub^, EXXXK^ub^, K^ub^D, K^ub^E and K^ub^A were extracted. Several Kub sites in ubiquitin-mediated proteolysis-related proteins, including RGLG2, UBC36, UEV1D, RPN10 and PSMC2, might affect protein degradation and DNA repair. Plenty of Kub proteins related to catechins biosynthesis, including PAL, CHS, CHI and F3H, were positively correlated with each other due to their co-expression and co-localization. Furthermore, some Kub proteins involved in carbohydrate and amino acid metabolism, including FBPase, FBA and GAD1, might promote sucrose, fructose and GABA accumulation in tea leaves under drought stress. Our study preliminarily revealed the global profiling of Kub proteins in metabolic pathways and provided an important resource for further study on the functions of Kub proteins in tea plants.

## Introduction

Tea (*Camellia sinensis* L.), as a kind of moisture loving plant, is always confronted with drought stress throughout its life cycle, and it has developed unique acclimation mechanisms that enhance its tolerance to drought stress^1^. A large number of drought-inducible genes and proteins in tea plants have been identified by transcriptome and proteome^2^. Many differentially expressed genes (DEGs) under drought stress were mainly involved in hormone biosynthesis, signal transduction and osmotic adjustment^3^. Many metabolites of tea leaves under drought stress were significantly decreased, such as catechins, caffeine, theanine, and free amino acids^4^. Our former study indicated that numerous DEGs were enriched in volatile compounds, flavonoid, theanine biosynthesis pathways and related to leaf senescence. And we found that the ubiquitin-26S proteasome system (UPS) was activated by drought stress^5^. Another study of proteome in tea leaves under drought stress indicated that plenty of proteins were involved in photosynthesis, sulfur-containing metabolite pathways, phenylpropanoid pathway. And the proteins related to ubiquitin-26S proteasomes were also identified and expressed differentially^6^. Several studies showed that UPS modulated the protein stability during drought stress. For example, the E3 ligase ZmAIRP4 (Zea mays Abscisic acid-Insensitive RING Protein 4) in *Arabidopsis* could enhance tolerance to drought stress^7^. The expression of E3 ligase AtRZF1 (RING Zinc Finger 1) in *Arabidopsis* was significantly influential in drought sensitive parameters and dehydration stress-related gene expressions^8^. CaPUB1 (*Capsicum annuum* Putative U-box protein 1), a hot pepper U-box E3 ligase in rice was more sensitive to water deficit and decreased tolerance to drought stress^9^.

Ubiquitination can mark proteins for degradation via the proteasome, alter protein subcellular location, affect their activities, and promote or inhibit protein interactions^10–12^. In plants, ubiquitination was associated with DNA damage response, membrane transport and transcriptional regulation, as well took part in enzymatic activity regulation and stress responses^13,14^. However, the role of ubiquitination in tea plants remains unearthed.

To investigate the possible mechanisms of Kub proteins in tea plants under drought stress, we studied the ubiquitome using antibody-based affinity enrichment coupled with LC-MS/MS analysis. Then, we analyzed the GO, KEGG and PPI of identified Kub proteins. Our study preliminarily revealed the global profiling of Kub proteins in metabolic pathways and provided an important resource for further study on the functions of Kub proteins in tea plants

## Results and Discussion

### Physiological characterization of tea plants subjected to drought and detection of Kub proteins in tea leaves

**Figure 1:**
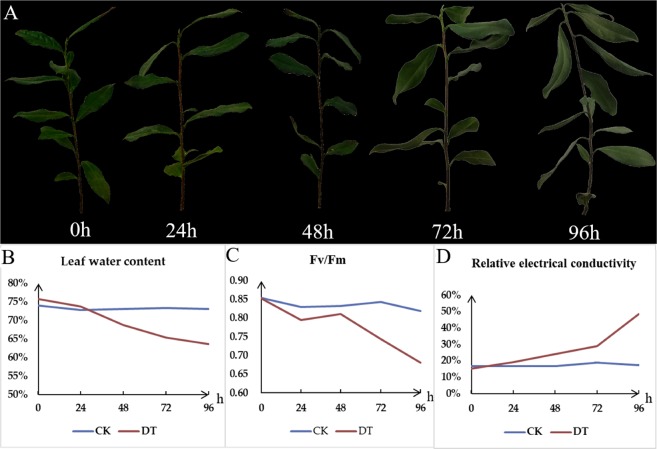
The morphological and physiological analyses of tea plants under drought stress. **(A)** The phenotypes of CK and DT, **(B)** leaf water content (LWC), **(C)** leaf maximum photochemical quantum yield of PS II (Fv/Fm), and **(D)** leaf relative electrolyte conductivity (REC) were determined at 5 time-points (0, 24, 48, 72, 96 hours).

To compare the impact of drought stress on physiological and plant responses, tea plants were exposed to drought from 0 h to 96 h. During drought stress, the morphology of tea leaves became wrinkled and shriveled, especially at 96 h. The REC, LWC, *Fv/Fm* of tea leaves were investigated (Fig. 1), the LWC and *Fv/Fm* were declined, while REC was increased in drought treatment (DT). The results indicated that drought stress really caused cell dehydration in tea leaves, resulting in damage of membrane and photosynthetic system at physiological level. In order to examine the expression patterns of the Kub proteins in tea leaves under drought stress, we performed Western Blotting Assay (Supplemental Fig. 1). The bands of multiple Kub proteins in control treatment (CK) and DT were detected and all proteins in DT were reduced by long induction of drought stress, suggesting that the Kub proteins in tea leaves were changed dynamically and the lysine-ubiquitinated peptides could be enriched by di-Gly-Lys-specific antibody.

### The ubiquitome profiles and functional classification of the Kub proteins in tea leaves under drought stress

In total, 1,409 Lys Kub sites were identified in 781 proteins, among which 1,226 sites were accurately quantified in 703 proteins. From these, 123 sites in 91 proteins were down-regulated and 14 sites in 12 proteins were up-regulated at a threshold of 1.5 (*p* < 0.05; Supplemental Table 1). Moreover, there were 70 Kub sites in 63 proteins identified in CK, including phenylalanine ammonia-lyase (PAL; Lys-93, Lys-335, Lys-337, Lys-523, Lys-554 and Lys-667), chalcone synthase 1 (CHS/TT4; Lys-181), chalcone–flavonone isomerase (CHI; Lys-96 and Lys-198) and leucoanthocyanidin reductase (LAR; Lys-66) (Supplemental Fig. 2; Supplemental Table 2). These Kub sites appeared in CK and disappeared after drought stress, suggesting that they were negatively regulated by drought stress. Meanwhile, 61 Kub sites in 52 proteins were identified in DT, including mannitol dehydrogenase (Lys-182), aconitate hydratase (Lys-864), fructose-1,6-bisphosphatase (FBPase; Lys-272) and fructose-bisphosphate aldolase (FBA; Lys-323), glutamate decarboxylase 1 (GAD1; Lys-5) and glutamine synthetase cytosolic isozyme 1 (GLN1-1; Lys-64 and Lys-65). These Kub proteins mainly related to carbohydrate metabolism and amino acid metabolism. The global proteome data were also collected with the identification of 4789 proteins (Supplemental Table 3).

**Figure 2:**
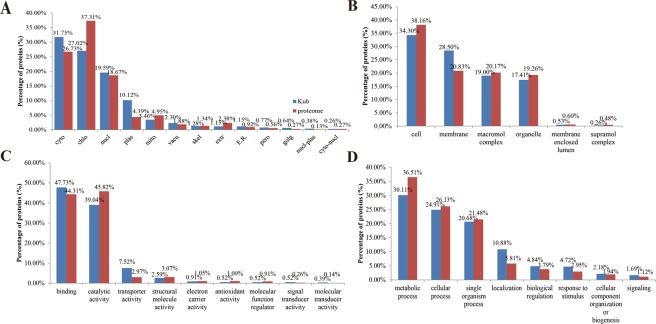
Subcellular localization and GO classification analysis of Kub protein compared to global proteome. (**A**) Subcellular localization, **(B)** cellular component, **(C)** molecular function and **(D)** biological process of Kub proteins compared to global proteome. Abbreviation, cyto for cytoplasm, chlo for chloroplast, nucl for nucleus, plas for plasma membrane, mito for mitochondria, vacu for vacuolar membrane, skel for cytoskeleton, extr for extracellular, E.R. for endoplasmic reticulum, pero for peroxisome and golg for golgi apparatus.

Subcellular localization of the identified Kub proteins was analyzed (Fig. 2A). Most of the Kub proteins were distributed in the cytosol (31.75%), chloroplast (27.02%), and nuclear (19.59%). The subcellular localization of whole proteome was also characterized for comparison (Fig. 2A). According to these data, the subcellular localization of Kub proteins and global proteome had few significant differences.

We also compared the GO classification of Kub proteins and global proteome, and found that both Kub proteins and global proteome showed similar patterns in cellular components, molecular functions and biological processes (Fig. 2B–D). In the cellular component, the Kub proteins mainly participated in cell and membrane. In the molecular function, the majority of Kub proteins were involved in binding and catalytic activity. In the biological process, the most of Kub proteins were associated with metabolic process and cellular process. These results indicated that lysine ubiquitome and proteome had similar localization distribution and biological functions.

### Functional enrichment of the Kub proteins

**Figure 3:**
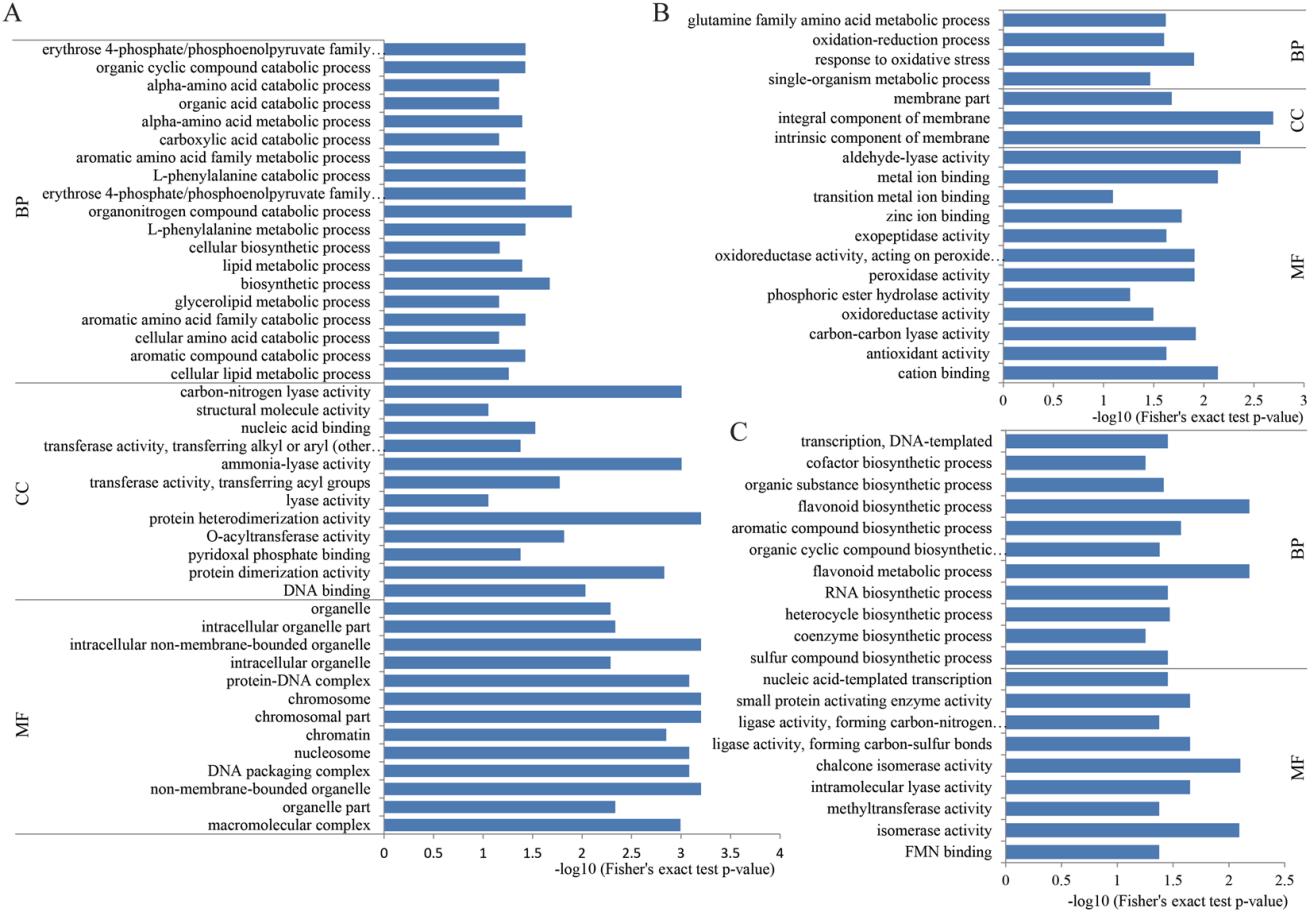
GO enrichment analysis of Kub proteins. (**A**) GO-based enrichment analysis of common Kub proteins between DT and CK, **(B)** DT-unique Kub proteins and **(C)** CK-unique Kub proteins. BP: biological process, CC: cellular component, MF: molecular function.

To better understand the functions of Kub proteins in tea leaves, GO functional enrichment of the identified Kub proteins was conducted (Supplemental Table 4). Some Kub proteins in both CK and DT were commonly enriched in biosynthetic process (e.g., O-acyltransferase WSD1-like, ubiquitin-60S ribosomal protein L40, 40S ribosomal protein S2-4, cellulose synthase-like protein E6, squalene synthase and CHS1), L-phenylalanine metabolic process (e.g., PAL1 and PAL) and chromosomal part (histone H3.2-like, histone H2AX probable histone H2A.1, histone H2A variant 1 and probable histone H2B.1) (Fig. 3A). But in DT, some DT-specific Kub proteins were enriched in membrane part (including tetraspanin-19, oleosin 1-like and patellin-3), response to oxidative stress (including catalase isozyme 3 and glutathione peroxidase 5) and glutamine family amino acid metabolic process (including GAD1 and GLN1-1) (Fig. 3B). Meanwhile, some DT-specific Kub proteins were enriched in aldehyde-lyase activity (including ketose-bisphosphate aldolase class-II family protein and fructose-bisphosphate aldolase cytoplasmic isozyme), cation binding (including RNF2 RING finger protein B, mannitol dehydrogenase and E3 ubiquitin-protein ligase RMA1H1), and exopeptidase activity (including prolyl endopeptidase and probable aspartyl aminopeptidase). In CK, many Kub proteins were uniquely enriched in chalcone isomerase activity (chalcone–flavonone isomerase 3, chalcone–flavonone isomerase 2), flavonoid metabolic process (chalcone synthase 1, naringenin,2-oxoglutarate 3-dioxygenase), and small protein activating enzyme activity **(**glutamine synthetase cytosolic isozyme 1, pyridoxal 5′-phosphate synthase subunit PDX1) (Fig. 3C). In addition, there were still 56 Kub proteins not enriched in any GO terms, including 9 CK-specific Kub proteins (including ubiquitin-conjugating enzyme E2 variant 1D, lipid phosphate phosphatase 2-like isoform X2, protein sieve element occlusion B-like and plant/F25P12-18 protein), 9 DT-specific Kub proteins (including DNA-binding family protein, heat shock cognate 70 kDa protein 2, putative methylesterase 14, fruit protein pKIWI501-like and UNC93-like protein 1) and 38 differentially expressed Kub proteins in common between CK and DT (including glycine-rich domain-containing protein 1-like, 101 kDa malaria antigen, protein plastid movement impaired 1, protein MLP1 isoform X2 and CASP-like protein 1). To our knowledge, these Kub proteins were first found in tea plants up to now. As for these Kub proteins, we need further identification.

**Figure 4:**
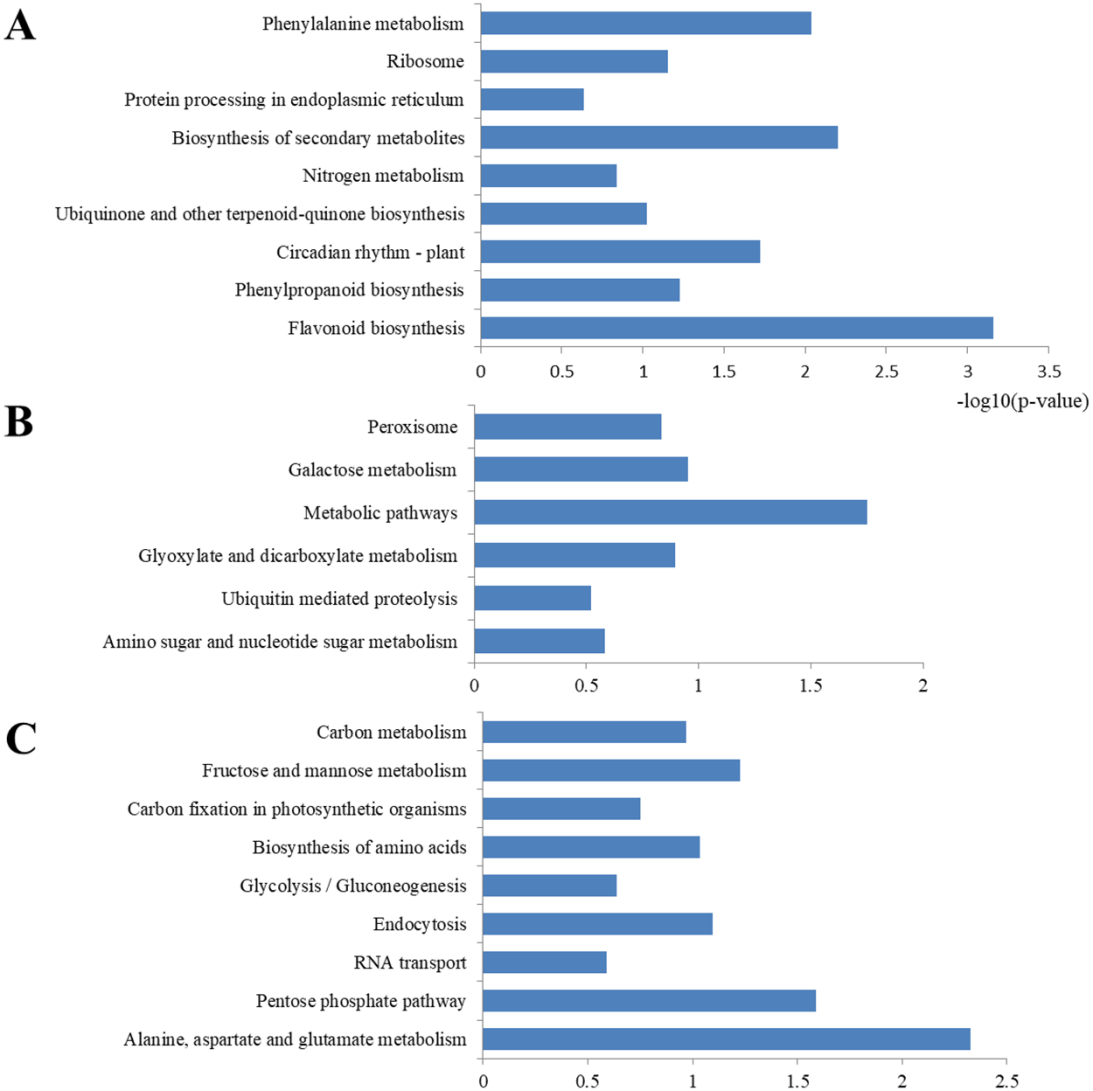
KEGG enrichment analysis of Kub proteins. (**A**) KEGG enrichment of common Kub proteins in CK and DT, **(B)** CK-unique Kub proteins and **(C)** DT-unique Kub proteins.

KEGG pathway analysis showed that most of the Kub proteins modulated in CK and DT were mapped to similar pathways (Supplemental Table 5; Fig. 4A), such as flavonoid biosynthesis (including CHS, LAR, naringenin,2-oxoglutarate 3-dioxygenase and flavonoid 3′ hydroxylase), phenylpropanoid biosynthesis (including trans-cinnamate 4-monooxygenaseand and 4-coumarate-CoA ligase 2), ribosome (including 60S ribosomal protein L11, 60S ribosomal protein L28-2-like, ubiquitin-60S ribosomal protein L40, 40S ribosomal protein S2-4 and 40S ribosomal protein S3a) and protein processing in endoplasmic reticulum (including 17.3 kDa class I heat shock protein-like, cell division cycle protein 48 homolog, heat shock cognate 70 kDa protein 2 and cell division cycle protein 48 homolog). However, many CK-unique Kub proteins were enriched in amino sugar and nucleotide sugar metabolism (including GDP-mannose 3,5-epimerase 2 and phosphoglucomutase), ubiquitin mediated proteolysis (including E3 ubiquitin-protein ligase UPL1 isoform X2 and NEDD8-activating enzyme E1 catalytic subunit isoform X1), galactose metabolism (including probable galactinol-sucrose galactosyltransferase 6 isoform X1 and phosphoglucomutase) and peroxisome (including peroxisomal (S)-2-hydroxy-acid oxidase GLO1 isoform X1) (Fig. 4B). Meanwhile, many DT-specific Kub proteins were mapped to RNA transport (including elongation factor 1-alpha and polyadenylate-binding protein 2-like), endocytosis (including ras-related protein RABA2a and heat shock cognate 70 kDa protein 2), fructose and mannose metabolism (including fructose-1,6-bisphosphatase and fructose-bisphosphate aldolase cytoplasmic isozyme), carbon metabolism (including catalase isozyme 3 and aconitate hydratase) and alanine, aspartate and glutamate metabolism (including glutamate decarboxylase 1 and glutamine synthetase cytosolic isozyme 1) (Fig. 4C). In total, the results indicated that Kub proteins in tea leaves under drought stress were mainly involved in flavonoid, carbohydrate and glutamine metabolism pathway.

### Motif analysis and protein interaction networks for Kub proteins in tea plant

**Figure 5:**
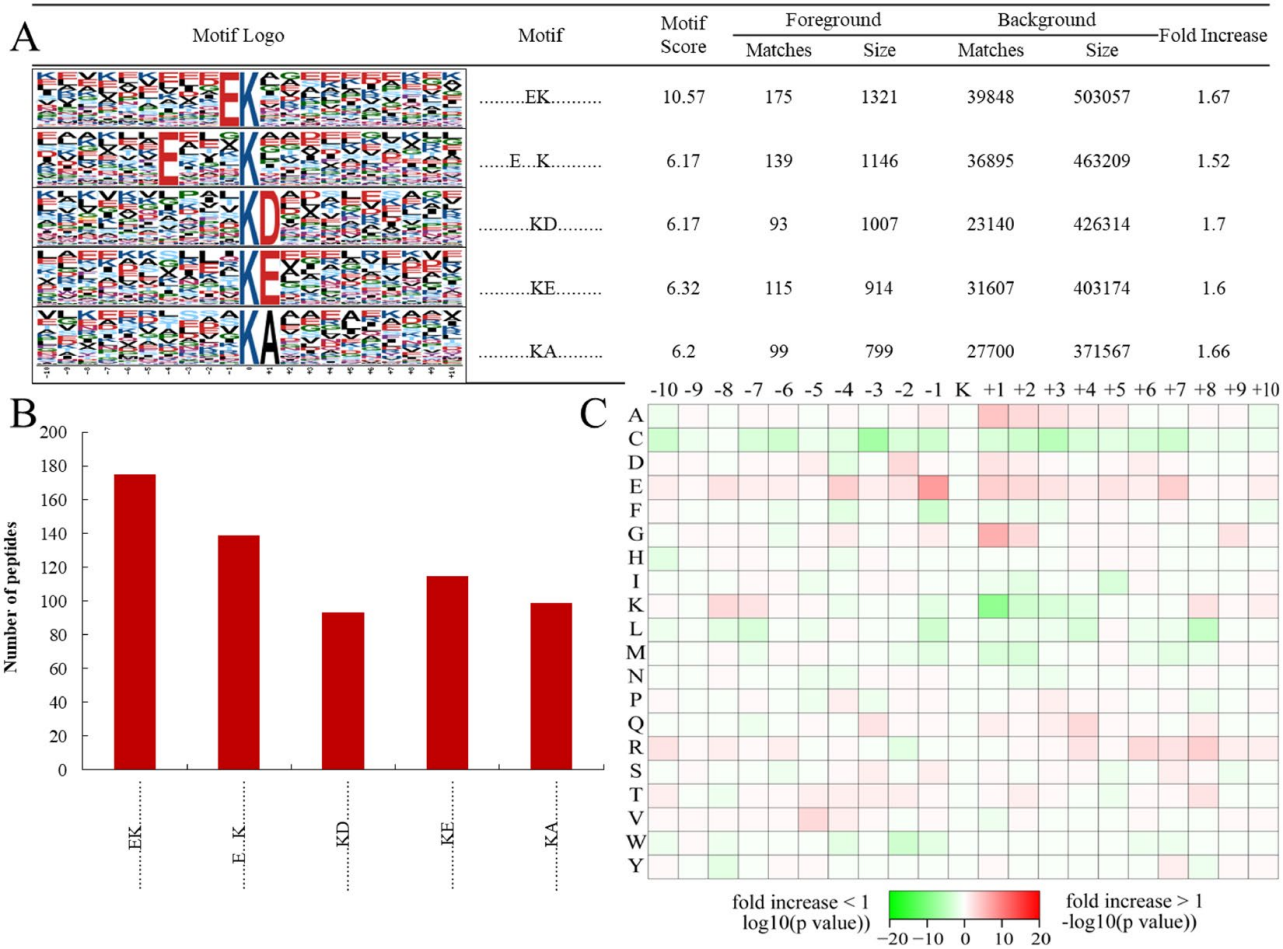
Motif analysis of all the identified Kub sites in tea leaves. (**A)** Ubiquitination motifs and the conservation of Kub sites. The height of each letter corresponds to the frequency of this amino acid residue in its position. The central K refers to the ubiquitinated Lys. **(B)** Number of identified peptides containing ubiquitinated Lys in each motif. **(C)** Amino acid sequence properties of ubiquitylation sites. The heat map showed significant position-specific underrepresentation or overrepresentation of amino acids flanking the modification sites.

In total, 622 of the 1409 Kub identified peptides were contained in amino acid sequences from −10 to +10 positions surrounding the Kub lysine. All of them were classified into 5 conserved motifs, including EK^ub^, EXXXK^ub^, K^ub^D, K^ub^E and K^ub^A (K^ub^ indicates the Kub lysine, and X indicates any amino acid), and they exhibited different abundances (Supplemental Table 6; Fig. 5). Among them, EK^ub^, EXXXK^ub^, K^ub^D and K^ub^E were reported as Kub motif in other published studies^15^, the K^ub^A was firstly reported in our study. Moreover, the Kub lysine motifs showed a strong preference for glutamic acid (E) in the −4, −1 and +1 positions, as well as for aspartic acid (D) and alanine (A) in the +1 position. Similar preference for amino acid residues, such as glutamic acid, aspartic acid and alanine, adjoining Kub Lys residues has been observed in petunia, wheat and rice^14–16^. These results indicated that different plants might share common conserved motifs surrounding Kub sites.

**Figure 6:**
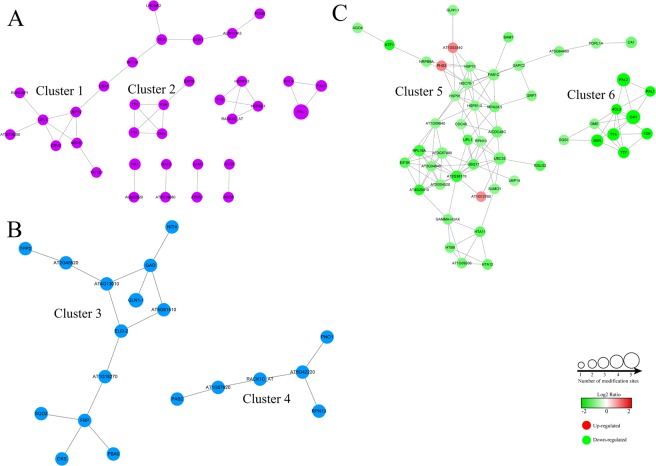
PPI networks of the Kub proteins in tea leaves under drought stress. Interaction networks of Kub proteins among (**A**) CK-unique proteins, (**B**) DT-unique proteins and (**C**) common proteins of CK and DT.

To predict relationships among the Kub proteins in different metabolic pathways, we generated protein-protein interaction (PPI) networks for CK-unique, DT-unique and their common proteins against the STRING database. Among CK-unique proteins, there were 34 Kub proteins mapped to the protein interaction networks (Supplemental Table 7; Fig. 6A). And they were clustered into 8 sub-networks. The most abundant sub-network (Cluster 1) consisted of 14 Kub proteins, including ubiquitin-activating enzyme E1 1 (UBA1), ubiquitin carboxyl-terminal hydrolase 12 (UBP12), DNA-directed RNA polymerase II subunit 1 (NRPB5) and ribulose bisphosphate carboxylase/oxygenase activase (RCA). The interactions of these Kub proteins were mediated by UBP12 which involved in ubiquitin process, indicating that ubiquitin might play an important role in their interactions. The second sub-network (Cluster 2) consisted of 5 catechins biosynthesis-related Kub proteins, including naringenin,2-oxoglutarate 3-dioxygenase (F3H), TT4, chalcone–flavonone isomerase 3 (CHIL) and chalcone–flavonone isomerase 2 (TT5), of which all of the proteins interacted with each other.

Among DT-unique proteins, there were 19 Kub proteins mapped to the protein interaction networks (Fig. 6B). And they were clustered into 2 sub-networks. The most abundant sub-network (Cluster 3) consisted of polysaccharide-associated proteins, in which 13 highly interconnected Kub proteins were retrieved, including FBPase, GAD, 3-deoxy-manno-octulosonate cytidylyltransferase (CKS), GLN1-1 and ketose-bisphosphate aldolase class-II family protein (AT1G18270). These protein interaction networks were centrally mediated by AT1G18270. The second sub-network (cluster 4) consisted of 6 Kub proteins, including polyadenylate-binding protein 2 (PAB2), peptide-N(4)-(N-acetyl-beta-glucosaminyl)asparagine amidase (PNG1), 26S proteasome regulatory subunit RPN13 (RPN13), guanine nucleotide-binding protein subunit beta (RACK1C), elongation factor 1-alpha (AT5G07920) and large proline-rich protein bag6-A (AT5G42220). The interactions of these Kub proteins were mediated by AT5G42220 which involved in proteasome binding process.

Among common proteins of CK and DT, there were 51 Kub proteins mapped to the protein interaction networks (Fig. 6C). The most abundant sub-network (Cluster 5) consisted of 41 Kub proteins, in which 10 highly interconnected UPS-associated Kub proteins were retrieved, including small ubiquitin-related modifier 1 (SUMO1), polyubiquitin isoform X1 (UBQ11), ubiquitin-conjugating enzyme E2 36 (UBC35/36), E3 ubiquitin-protein ligase RGLG2 (RGLG2), ubiquitin carboxyl-terminal hydrolase 14 (UBP14) and E3 ubiquitin-protein ligase At1g12760 (AT1G12760). And these interaction networks were mediated by UBC35. The second sub-network (Cluster 6) consisted of 10 catechins biosynthesis-related proteins, including PAL1, C4H, 4CL2, F3H and anthocyanidin reductase (BAN/ANR), of which many Kub proteins related to catechins biosynthesis interacted with C4H. The protein-protein interaction results suggested that lysine ubiquitination is relatively active in UPS, catechins biosynthesis, carbohydrate and amino acid metabolism in tea leaves.

### The expressions of Kub proteins involved in ubiquitin-proteasome system under drought stress

**Figure 7:**
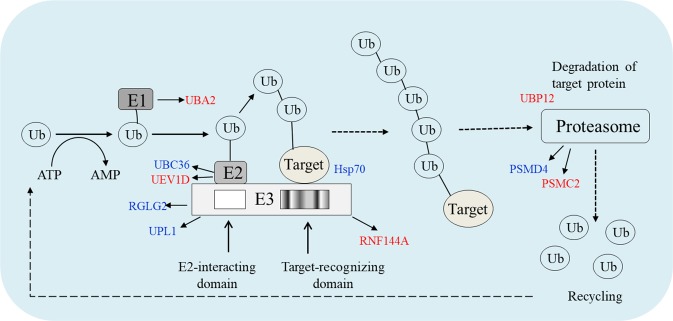
Proteolysis pathway was mediated by ubiquitin in tea leaves under drought stress. The blue words indicate down-regulated proteins under drought stress, and the red words indicate proteins only identified in CK.

To demonstrate the expression of Kub proteins involved in proteolysis, we illustrated the process of UPS (Fig. 7). The UPS selectively removes substrate proteins by labeling ubiquitin protein tags and the activity of a series of enzymes. In the results, 14 Kub sites in 11 UPS-related proteins of CK and DT were significantly down-regulated under drought stress, such as Lys-29 and Lys-135 in RGLG2, Lys-118, Lys-191 and Lys-144 in UBC36, and Lys-74 in 26S proteasome non-ATPase regulatory subunit 4 (RPN10). Moreover, 6 Kub sites were found in 6 CK-unique proteins, including Lys-13 in 26S proteasome regulatory subunit 7 (PSMC2) and Lys-136 in ubiquitin-conjugating enzyme E2 variant 1D (UEV1D).

Previous research indicated that RGLG2, the RING domain ubiquitin E3 ligase, was negatively regulated by drought stress in *Arabidopsis* and a single mutant seedling, of *rglg2* exhibited a dehydration-tolerant phenotype^17^. Moreover, RGLG2 catalyzed the synthesis of Lys-63-linked multiubiquitin chains^18^. Meanwhile, the Ubc13-Uev heterodimer consisted of Ubc13 and Uev was also required for the formation of Lys-63 linked multiubiquitin chains^19^. Since the multiubiquitin chains were involved in several cellular processes, including signal transduction, stress response and DNA repair^20,21^, RGLG2, Ubc13 and Uev might be involved in these cellular processes. The down-regulation of Kub sites in RGLG2, UBC36 (homolog of Ubc13) and UEV1D (Uev enzyme variant) suggested that the formation of Lys-63 linked chains might be inhibited under drought stress. So, it is tempting to speculate that RGLG2, UBC36 and UEV1D may play important roles in signal transduction and DNA repair.

26S proteasome consisted of multiple protein components catalyzes ATP-dependent breakdown of proteins conjugated with ubiquitin. The proteasome participated in several biological processes, including cell cycle progression, apoptosis, or DNA damage repair^22^. RPN10, the subunit of 26S proteasome, acted as an ubiquitin acceptor subunit through ubiquitin interactions and selected ubiquitin-proteins for destruction in human^23^. In *Arabidopsis*, RPN10 increased 20S proteasome levels which degraded proteins into small peptides, and thus enhanced Ub-independent protein degradation^24^. However, there is no report about the RPN10 in plants under drought stress. The down-regulation of Kub site in RPN10 in our study indicated that drought stress, to some extent, might enhance Ub-independent protein degradation. In addition, PSMC2, another subunit of 26S proteasome, could translocate Kub target proteins into a proteolytic chamber and degrade them into peptides in human and animals (such as mouse, bovine and rat)^25^. However, the functions of PSMC2 in plants were seldom reported. In the present study, the Kub site in PSMC2 of tea leaves was down-regulated under drought stress. We speculate that the down-regulation of Kub site in RPN10 and PSMC2 under drought stress may facilitate degradation of Kub protein, as well participate in cell cycle progression, apoptosis, or DNA damage repair in tea leaves.

### The expressions of Kub proteins related to catechins biosynthesis under drought stress

**Figure 8:**
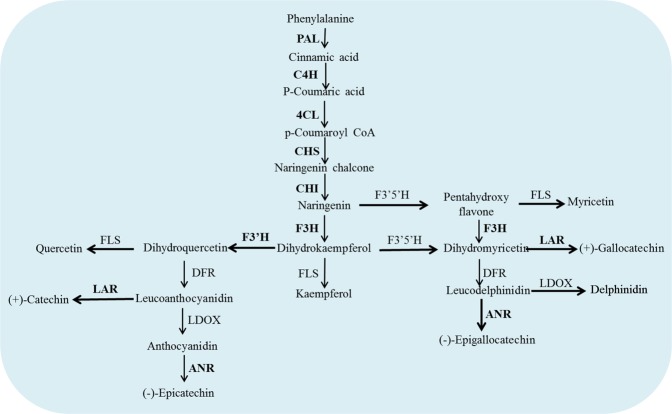
Proteins in catechins biosynthesis pathway in tea leaves were ubiquitinated under drought stress. The boldfaces indicate the specific expression Kub proteins under drought stress.

To elucidate the influence of Kub proteins related to catechins biosynthesis under drought stress, we analyzed the expressions of Kub proteins involved in phenylpropanoid and flavonoid pathway (Fig. 8). In the results, 31 Kub sites in 11 common proteins of CK and DT were significantly down-regulated, such as Lys-37, Lys-98, Lys-113, Lys-120, Lys-326, Lys-383 and Lys-622 in PALs, Lys-83, Lys-108, Lys-161, Lys-288 and Lys-447 in C4H, Lys-233, Lys-240, Lys-335, Lys-378 and Lys-381 in CHS, and Lys-146 in 4CL. Moreover, there were 15 Kub sites in 9 CK-unique proteins, including Lys-96 in CHIL and Lys-382 in F3H.

PAL is the first and committed step in the phenylpropanoid pathway. It is involved in the biosynthesis of the polyphenol compounds. The ubiquitination of PAL in petunia negatively correlated with the expression of PAL under ethylene treatment^15^. The content of catechins decreased under drought stress, which was consistent with the expression of PAL^26^. PAL was up-regulated in slight drought and down-regulated in serious drought^6^. The data in our study showed that the contents of ECG, EGCG in tea leaves increased under drought stress (Supplemental Table 8), but the Kub sites in PAL were all down-regulated. Therefore, we speculate that the UPS degraded PAL by ubiquitination and the down-regulation of PAL may negatively regulated biosynthesis of catechins under drought stress.

The enzyme CHS catalyzes the condensation of 4-hydroxycinnamoyl CoA and malonyl-CoA to form chalcone, which is the substrate for CHI and convert to naringenin. *CHS* and *CHI* were the critical genes in regulating catechins contents in tea plants in response to drought^27^. The contents of EGCG and total catechins had significant positive correlations with *CHS* and *CHI* during the development of tea leaves^28^. Moreover, CHS and CHI were detected being acetylated and differentially accumulated in leaves of ‘Anjin Baicha’ (an albino tea cultivar), suggesting that this PTM (post-translational modification) may contribute to the abundance of flavonoid across the developmental stages^29^. However, little research has been devoted to the ubiquitination of CHS and CHI in tea plants under drought stress. In present study, the content of naringenin was decreased and the Kub sites in CHS and CHI were down-regulated in tea leaves under drought stress, suggesting that the expression of Kub sites in CHS and CHI might positively regulate in the biosynthesis of naringenin. But the content of EGCG in tea leaves was increased under drought stress. Therefore, we speculate that the biosynthesis of EGCG may negatively associate with the expression of Kub sites in CHS and CHI in response to drought. Furthermore, an evidence showed that CHS and CHI were co-localized at the endoplasmic reticulum and tonoplast in *Arabidopsis* and the expressions of CHS and CHI were consistent with the higher accumulation of flavonoids^30^. In prior research, CHIL (type IV CHI protein) co-expressed, co-localized, and interacted with CHI for flavonoid production in *Arabidopsis*^31^. Previous study showed that CHS and CHI interacted with F3H and assembled as a macromolecular complex to promote flavonoid production in *Arabidopsis*, and CHI was posttranslationally modified, which played a role in controlling the association of CHI with other flavonoid enzymes^32^. However, to our knowledge, there is no report about the role of Kub CHS and CHI in plants. In our results, CHS, CHI, CHIL and F3H were all ubiquitinated in tea leaves and all the Kub sites in these proteins were down-regulated under drought stress. The PPI analysis showed that the interactive relationships existed among all of the four Kub proteins, suggesting that the down-regulation of 4 proteins accordingly slowed the reaction down from malonyl-CoA to dihydrokaempferol under drought stress. Therefore, we speculate that ubiquitination might play a key role in the interaction of CHI, CHS, CHIL and F3H. Their common down-regulations might decrease the flavonoid production.

### The expressions of Kub proteins related to carbohydrate and amino acid metabolism under drought stress

To elucidate the Kub proteins participated in carbohydrate and amino acid metabolism induced by drought stress, we analyzed the DT-unique Kub proteins in tea leaves. There were 6 Kub sites in 6 DT-unique proteins related to carbohydrate metabolism, including Lys-182 in GAD1, Lys-864 in aconitate hydratase, Lys-323 in FBA, and Lys-272 in FBPase. These Kub proteins were mainly involved in fructose and sucrose metabolism. Moreover, there were 3 Kub sites in 2 DT-unique proteins related to glutamate metabolism, including Lys-5 in GAD1, and Lys-64, Lys-65 in GLN1-1.

As for FBPase, three different groups of FBPase have been identified in eukaryotes and bacteria^33^. FBPase increased the soluble sugar (sucrose, glucose, and fructose) levels in the leaves of *Arabidopsis*, suggesting that the simultaneous overexpression of FBPase enhanced source capacity and consequently led to growth enhancement in transgenic plants^34^. High levels of FBPase contributed to the conversion of hexose into sucrose in tobacco, indicating that the increased FBPase activity led to enhance the synthetic ability and translocation efficiency of sucrose from source leaves to sink leaves^35^. Moreover, several metabolic enzymes involved in the Calvin cycle in wheat, including FBA, Rubisco, FBPase, and GAP, were found to be ubiquitinated^16^. However, no work has been done on the ubiquitination of FBPase in plants under drought stress. In the present research, the Kub site in FBPase induced by drought stress might promote sucrose accumulation.

FBA is an important enzyme in plants which is involved in glycolysis and the Calvin cycle, and plays a significant role in different stress responses. The overexpression of FBA increased the expressions of other main enzymes in Calvin cycle, net photosynthetic rate under salt stress, suggesting that FBA controlled photosynthesis, carbon partitioning and plant growth in tomato^36^. And, the abundance of FBA was increased under drought stress in *Eragrostis tef*, suggesting that drought stress may function in fructose-6-phosphate generation^37^. Furthermore, FBA involved in glycolysis and photosynthesis was detected to be ubiquitinated in rice, and was implied have a close relationship with salt tolerance^14^. In our study, the inducible expression of Kub site of FBA by drought stress indicated that the Kub site of FBA might regulate fructose biosynthesis in tea leaves under drought stress.

In addition, GAD1, the main enzyme of GABA biosynthesis, was involved in feedback controls of Ca^2+^-permeable channels to fluctuate intracellular GABA levels in tobacco, suggesting that GAD1 activity linked with Ca^2+^-permeable channels relayed an extracellular GABA signal^38^. And, the up-regulation of *GAD* gene expression induced the increased level of GABA under chlorsulfuron treatments in wheat, suggesting that GABA molecule might act as a protective and metabolic signaling molecule in carbohydrate and amino acid metabolism in plants under herbicidal treatments^39^. Moreover, the GABA level was increased in the *gad1/2* mutant of *Arabidopsis* seedling under drought stress, and GABA accumulation during drought regulated the stomatal opening and prevented loss of water^40^. To our knowledge, little attention has been paid on the ubiquitination of GAD1 in plants under drought stress. Our study showed that the Kub site of GAD1 in tea leaves was induced by drought stress, suggesting that the Kub GAD1 might involve the GABA metabolism in tea leaves under drought stress.

## Conclusion

In this study, we performed a global profile of Kub proteins in tea leaves under drought stress. Our results revealed that a large number of Kub proteins in tea leaves were participated in metabolic pathways, including ubiquitin-mediated proteolysis, catechins biosynthesis, and carbohydrate and amino acid metabolism. Several Kub sites in ubiquitin-mediated proteolysis-related proteins were down-regulated under drought stress, including RGLG2, UBC36, UEV1D, RPN10 and PSMC2. These Kub proteins might affect protein degradation and DNA repair in tea leaves. A large number of Kub proteins were related to catechins biosynthesis, including PAL, CHS, CHI and F3H, suggesting that these proteins were positively correlated with each other due to their co-expression and co-localization. Furthermore, some Kub proteins involved in carbohydrate and amino acid metabolism, including FBPase, FBA and GAD1, were induced significantly by drought stress, suggesting that these Kub proteins might promote sucrose, fructose and GABA accumulation in tea leaves. Based on this study, we can conclude that these proteins are indeed modified by ubiquitin, but as to the molecular outcome for each of these events it remains to be determined. Our study preliminarily revealed the roles of Kub proteins in metabolic pathways and provided an important resource for the further study of Kub functions in tea plants in response to drought stress.

## Materials and Methods

### Plant materials and stress treatments

Two-year old tea plants, *C. sinensis* (L.) O. Kuntze cv. “Zhongcha108”, were culture-grown under a 12 h light (25 °C)/12 h dark (20 °C) photoperiod with 1800 Lx photos m^−2^·s^−2^ light intensity and 75% humidity in growth chamber for 2 weeks^5^. Tea plants were divided into two groups: well-watered plants were irrigated every day (CK) and the other plants were dried until 96 hours (DT).

### Physiological experiments and protein extraction

Tea plants (three biological replicates) were collected for DT and CK at 0, 24, 48, 72 and 96 hours. The relative electrolytic conductivity (REC), leaf water content (LWC) and leaf maximum photochemical quantum yield of PS II (Fv/Fm) of different treatment were determined as described previously^14^.

Tea leaves (1 g/fw) were grinded into cell powders by liquid nitrogen and then transferred to a 5-mL centrifuge tube. After that, four volumes of lysis buffer (8 M urea, 1% Triton-100, 10 mM dithiothreitol and 1% Protease Inhibitor Cocktail, and 3 μM TSA and 50 mM NAM inhibitors) were added to the cell powders, followed by sonication on ice using a high intensity ultrasonic processor (Scientz). The remaining debris was removed by centrifugation at 20,000, 4 °C for 10 min. Finally, the protein was precipitated with cold Trichloroacetic acid (TCA) (supernatant/TCA, 17:3, v/v) at −20 °C for. After centrifugation at 12,000 g, 4 °C for 10 min, the supernatant was discarded. The remaining precipitate was washed with cold acetone for three times. The protein was redissolved in 8 M urea and the protein concentration was determined with BCA kit according to the manufacturer’s instructions.

### Trypsin digestion

For digestion, 4 mg of isolated protein solution was reduced with 5 mM dithiothreitol for 30 min at 56 °C and alkylated with 11 mM iodoacetamide for 15 min at room temperature in darkness. The protein sample was then diluted by adding 100 mM NH_4_HCO_3_ to urea concentration less than 2 M. Finally, trypsin was added at 1:50 trypsin-to-protein mass ratios for the first digestion overnight and 1:100 trypsin-to-protein mass ratios for a second 4 h-digestion.

### Tandem mass tag labeling

After trypsin digestion, peptide was desalted with a Strata X C18 SPE column (Phenomenex) and vacuum dried. Peptide was reconstituted in 0.5 M TEAB and processed according to the manufacturer’s protocol for the six-plex Tandem Mass Tag (TMT) kit. Briefly, 1 unit of TMT reagent (defined as the amount of reagent required to label 100 mg of protein) was thawed and reconstituted in 24 mL of acetonitrile. The peptide mixtures were then incubated for 2 h at room temperature and pooled, desalted, and dried by vacuum centrifugation.

### HPLC fractionation

The tryptic peptides were fractionated into fractions by high pH reverse-phase HPLC using Agilent 300 Extend C18 column (5 μm particles, 4.6 mm ID, 250 mm length). Briefly, peptides were first separated with a gradient of 8% to 32% acetonitrile (pH 9.0) over 60 min into 60 fractions. Then, the peptides were combined into 18 fractions and dried by vacuum centrifuging.

### Affinity enrichment

To enrich Kub modified peptides, 4 mg tryptic peptides dissolved in NETN buffer (100 mM NaCl, 1 mM EDTA, 50 mM Tris-HCl, 0.5% NP-40, pH 8.0) were incubated with pre-washed 20 uL di-Gly-Lys antibody beads (PTMScan ubiquitin remnant motif K-ε-GG kit, Cell Signaling Technology) at 4 °C overnight with gentle shaking. Then the beads were washed four times with NETN buffer and twice with H_2_O. The bound peptides were eluted from the beads with 0.1% trifluoroacetic acids (TFA). Finally, the eluted fractions were combined and vacuum-dried. For LC-MS/MS analysis, the resulting peptides were desalted with C18 ZipTips (Millipore) according to the manufacturer’s instructions.

### LC-MS/MS analysis

The tryptic peptides were dissolved in 0.1% formic acids (solvent A), directly loaded onto a home-made reversed-phase analytical column (15-cm length, 75 μm i.d.). The gradient was comprised of an increase from 6% to 23% solvent B (0.1% formic acids in 98% acetonitrile) over 26 min, 23% to 35% in 8 min and climbing to 80% in 3 min then holding at 80% for the last 3 min, all at a constant flow rate of 400 nL/min on an EASY-nLC 1000 UPLC system.

The peptides were subjected to NSI source followed by tandem mass spectrometry (MS/MS) in orbitrap fusion™ tribrid (Thermo) coupled online to the UPLC. Intact peptides were detected in the orbitrap at a resolution of 60,000. Peptides were selected for MS/MS using NCE setting as 35 and ion fragments and detected in the Ion Trap. A top speed data-dependent procedure that alternated between one MS scan followed by most intense MS/MS scan was applied for the precursor ions above threshold intensity greater than 5E3 in the MS survey scan with 30.0 s dynamic exclusion. The electrospray voltage applied was 2.0 kV. Automatic gain control (AGC) was used to prevent overfilling of the ion trap; 1E4 ions were accumulated for generation of MS/MS spectra. For MS scans, the m/z scan range was 350 to 1550. Fixed first mass was set as 100 m/z. The mass spectrometry proteomics data are available via ProteomeXchange with identifier PXD011688. The proteomics for TMT experiment corresponds to 1–18 raw files, representing 18 fractions. And the raw file of ubiquitination modification was named by the respective sample.

### Database search

The resulting MS/MS data were processed using Maxquant search engine (v.1.5.2.8). Tandem mass spectra were searched against Tea tree Genome database concatenated with reverse decoy database. During the database search, the modifications were set as follows: main search ppm: 6; missed cleavage: 4; MS/MS tolerance ppm: 20; De-Isotopic: TRUE; enzyme: trypsin; fixed modification: carbamidomethyl (C); variable modification: oxidation (M), acetyl (Protein N-term), GlyGly (K); decoy database pattern: reverse; iBAQ: TRUE; match between runs: 2 min; minimum peptide length: 7; false discovery rate (FDR) thresholds for proteins, peptides and modification sites: 0.01.

### Bioinformatics analysis

Bioinformatics analysis was performed according to previously described protocols^14^. The Gene Ontology (GO) annotation proteome was derived from the UniProt-GOA database (http://www.ebi.ac.uk/GOA/)^41^. The lysine ubiquitination peptide ID was converted to a UniProt ID and then mapped to a GO ID. The Kub proteins were then further classified by GO annotation based on three categories: biological processes, molecular functions, and cellular components. A two-tailed Fisher’s exact test was employed to test the enrichment of the differentially expressed protein against all identified proteins. The Kyoto Encyclopedia of Genes and Genomes (KEGG) database was used to annotate protein pathways^42^. The KEGG online service tool KAAS was used to annotate the proteins’ KEGG database description. The annotation results were mapped on the KEGG pathway database using the KEGG online service tool KEGG Mapper. The domain annotation was performed with InterProScan on the InterPro domain database via web-based interfaces and services. WoLF PSORT was used for predicting the subcellular localization^43^. The CORUM database was used to annotate protein complexes.

Motif-X software was used to analyze the model of the sequences with amino acids in specific positions of ubiquityl-15-mers (seven amino acids upstream and downstream of the Kub site) in all of the protein sequences. In addition, all the database protein sequences were used as the background database, and the other parameters were set to the default values. The setting parameters for searching motifs using Motif-X software was “occurrences 20” and “the Bonferroni corrected *p* = 0.005”.

All CK-unique, DT-unique and their common DEPs were selected for protein-protein interactions. First, the sequences of these DEPs were fetched and then upload to the STRING (http://string-db.org/). Next, the model species *Arabidopsis thaliana* was selected as a database. Through BLAST, the DEPs were matched to the Arabidopsis protein. STRING defines a metric called “confidence score” to define interaction confidence. The interactions that confidence score ≥0.4 were selected. Finally, interaction networks from STRING were visualized with Cytoscape^44^.

### Western blotting

Proteins were separated using SDS-PAGE (10% acrylamide gels) and blotted onto nitrocellulose membranes (BA-S 85; Schleicher & Schuell). The membrane was blocked with 5% skim milk powder and TBS (50 mM Tris-HCl, pH 8, and 150 mM NaCl) at room temperature for 1 hour. Purified ubiquitin antibody (PTM-1106: Lot: Z261E317P2; 1:1000 dilution) was used at a concentration of 50 mg mL^−1^. The membrane was washed three times with TBST (composed of TBS and Tween-20) for 10 minutes each time and then reacted with horseradish peroxidase-conjugated goat anti-rabbit IgG (Pierce) at a dilution of 1:5,000 (TBST containing 5% skim milk powder). Detection was achieved using WBKLS0500 (Millipore).

## Supplementary information

Dataset

## Data Availability

The datasets generated during the current study are available in the [ProteomeXchange] repository, [http://www.ebi.ac.uk/pride].
