# Supplementary material for: Global Ubiquitome Profiling Revealed the Roles of Ubiquitinated Proteins in Metabolic Pathways of Tea Leaves in Responding to Drought Stress
Source: Sci Rep. 2019 Mar 12;9:4286. doi: 10.1038/s41598-019-41041-3 (PMC6414630; doi:10.1038/s41598-019-41041-3)
Supplement: Supplementary file 1 — Dataset [file 41598_2019_41041_MOESM1_ESM.zip › Supplemental Figure 1-2.docx]

Global Ubiquitome Profiling Revealed the Roles of Ubiquitinated Proteins in Metabolic Pathways of Tea Leaves in Responding to Drought Stress

Hui Xie^1^, Yu Wang^1^, Yiqian Ding^1^, Chen Qiu^1^, Litao Sun^1^, Zhongshuai Gai^2^, Honglian Gu^1^, Zhaotang Ding^1^*

1 Tea Research Institute, Qingdao Agricultural University, Qingdao, 266109, China

2 College of Life Science, Yantai University, Yantai，Shandong, 264005, China

* Corresponding author. Tea Research Institute, Qingdao Agricultural University,

NO. 700 Changcheng Road, Qingdao, 266109, China.

E-mail address: dzttea@163.com (Z. Ding).


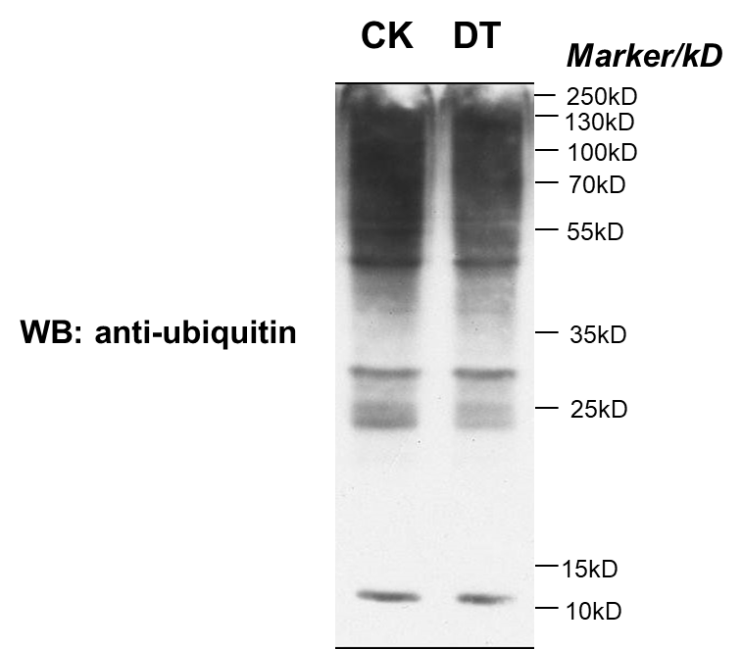


Fig. S1 Effects of drought stress on the expression of ubiquitin in tea plant in protein level. The proteins were detected by western blot analysis using an anti-ubiquitin antibody, and the full-length blots are included.

**
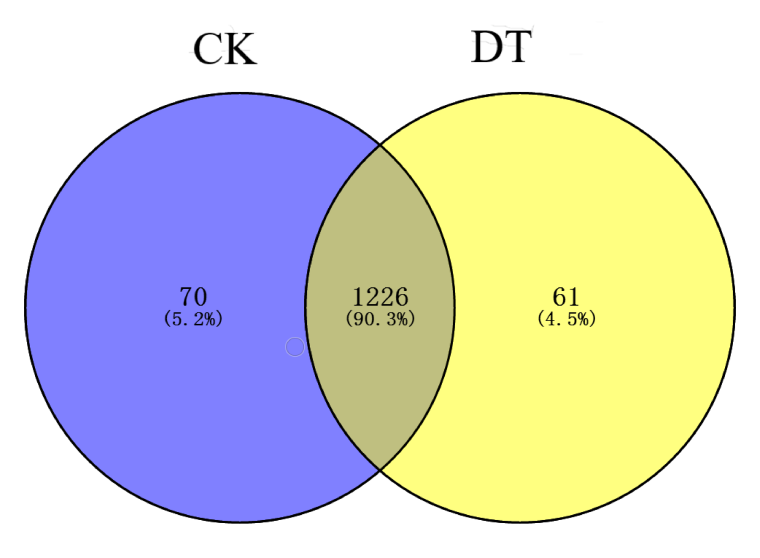
**

Fig. S2 Venn diagram illustrating the Kub sites from drought and control samples.
